# Effects of integrated biocontrol on bacterial wilt and rhizosphere bacterial community of tobacco

**DOI:** 10.1038/s41598-021-82060-3

**Published:** 2021-01-29

**Authors:** Yun Hu, Yanyan Li, Xiaoqiong Yang, Chunli Li, Lin Wang, Ji Feng, Shouwen Chen, Xihong Li, Yong Yang

**Affiliations:** 1 State Key Laboratory of Biocatalysis and Enzyme Engineering, School of Life Science, Hubei University, Wuhan, 430062 China; 2 Tobacco Research Institute of Hubei Province, Wuhan, 430030 China; 3 Hubei Tobacco Industry Co., Ltd., Wuhan, 430040 China

**Keywords:** Soil microbiology, Microbial communities, Microbe

## Abstract

Bacterial wilt as a soil-borne disease was caused by *Ralstonia solanacearum*, and seriously damages the growth of tobacco. Integrated biocontrol method was explored to control bacterial wilt. Nevertheless, the long-term effects of the integrated biocontrol method on soil bacterial community, soil physicochemical properties and the incidence of bacterial wilt are not well understood. In this study, *B. amyoliquefaciens* ZM9, calcium cyanamide and rice bran were applied to tobacco fields in different ways. The disease index and incidence of tobacco bacterial wilt (TBW), soil physicochemical properties, colonization ability of *B. amyoliquefaciens* ZM9, and rhizopshere bacterial community were investigated. The results showed that the integrated application of *B. amyoliquefaciens* ZM9, rice bran and calcium cyanamide had the highest control efficiency of TBW and bacteria community diversity. Additionally, the integrated biocontrol method could improve the colonization ability of *B. amyoliquefaciens* ZM9. Furthermore, the integrated biocontrol method could effectively suppress TBW by regulating soil physicochemical properties, promoting beneficial bacteria and antagonistic bacteria of rhizopshere soil. This strategy has prospect of overcoming the defects in application of a single antagonistic bacteria and provides new insights to understand how to improve the colonization capacity of antagonistic bacteria and control efficacy for TBW.

## Introduction

Bacterial wilt is a typical soil-borne disease caused by *Ralstonia solanacearum*^1^. Due to the high lethality and wide host range, it is widely distributed worldwide, which seriously affects the yield and quality of crops^2,3^. Some researchers believed that the rhizosphere microecological imbalance was the main reason for bacterial wilt^4^. The interdependent ecosystem formed by plants soil microorganisms was destroyed, while the imbalance of rhizosphere microecological further deteriorated the soil microenvironment, unhealthy growth of plants, a large number of pathogenic bacteria multiplying, and eventually lead to outbreaks of soil-borne diseases^5,6^.

Biological control is widely used for prevention and control of soil-borne diseases due to its broad-spectrum, persistent and environmentally friendly characteristics^7,8^. The most common method of biological control is to control the abundance of pathogenic bacteria through antagonistic bacteria, so as to reduce the harm of pathogens to plants, and antagonistic bacteria are also beneficial to plant growth^9,10^. Until now more than 100 antagonists of bacterial wilt include *Bacillus* spp., *Pseudomonas* spp., *Streptomyces* spp. and so on were isolated and identified^11,12^. However, in practical application, these antagonists were affected by environmental conditions and crops. Moreover, the control efficacy is unstable, the adaptability to the environment is poor, and the colonization ability is decreased^13^. Since the colonization of antagonistic in soil is difficult after the application of antagonistic bacteria alone, the improvement of the colonization of antagonistic bacteria in the soil rhizosphere has become the research focus of biological control. Previous studies demonstrated that antagonistic bacteria could better colonize plant roots when they were applied to the soil with organic fertilizers or soil amendments^14,15^. At the same time, plant root exudates also have a positive induction effect on microbial biofilm formation and chemotactic, thus inhibiting the growth of pathogenic bacteria or improving plant resistance^16,17^. Therefore, organic fertilizers or soil amendments assistance of antagonistic bacteria should be considered as an effective integrated biocontrol method to improve the colonization ability of antagonistic bacteria and control bacterial wilt. However, most trials about the integrated biocontrol have been carried over short time periods^18^. Since the quality of the soil, such as the physicochemical properties, microbial community, etc. is extremely important for the growth of plants, changing the physicochemical properties of the soil is a long process that could take several years. However, the long-term effects of the combined application of antagonistic bacteria, organic fertilizers or soil amendments on soil bacterial community, soil physicochemical properties and the incidence of bacterial wilt had been poorly understood in field experiment^15^.

Our previous study showed that *B. amyloliquefaciens* ZM9 as an antagonistic bacterium of bacterial wilt was isolated through its ability to colonize tobacco and rhizosphere soil, which can improve the microbial abundance of rhizosphere soil^5^. Studies have evidenced soil amendment calcium cyanamide can improve soil ecological environment, and increase crop yield^19,20^. Additionally, rice bran, as an effective organic fertilizer, provides soil microorganisms with carbon, nitrogen and other basic nutrients^21^. In this study, the experiment were carried out in infected tobacco fields for three years, and antagonistic bacteria *B. amyloliquefaciens* ZM9, rice bran and calcium cyanamide were applied to the fields in different ways. The main aims of this study were to (1) compare the control efficacy of different treatment groups for TBW, (2) assess the colonization ability of *B. amyloliquefaciens* ZM9, and (3) explore the dynamic changes of physicochemical properties and rhizosphere bacterial community. To provide new insights to understand how to improve the colonization ability of antagonistic bacteria and control efficacy for TBW.

## Results

### Control of TBW by different treatments

**Figure 1:**
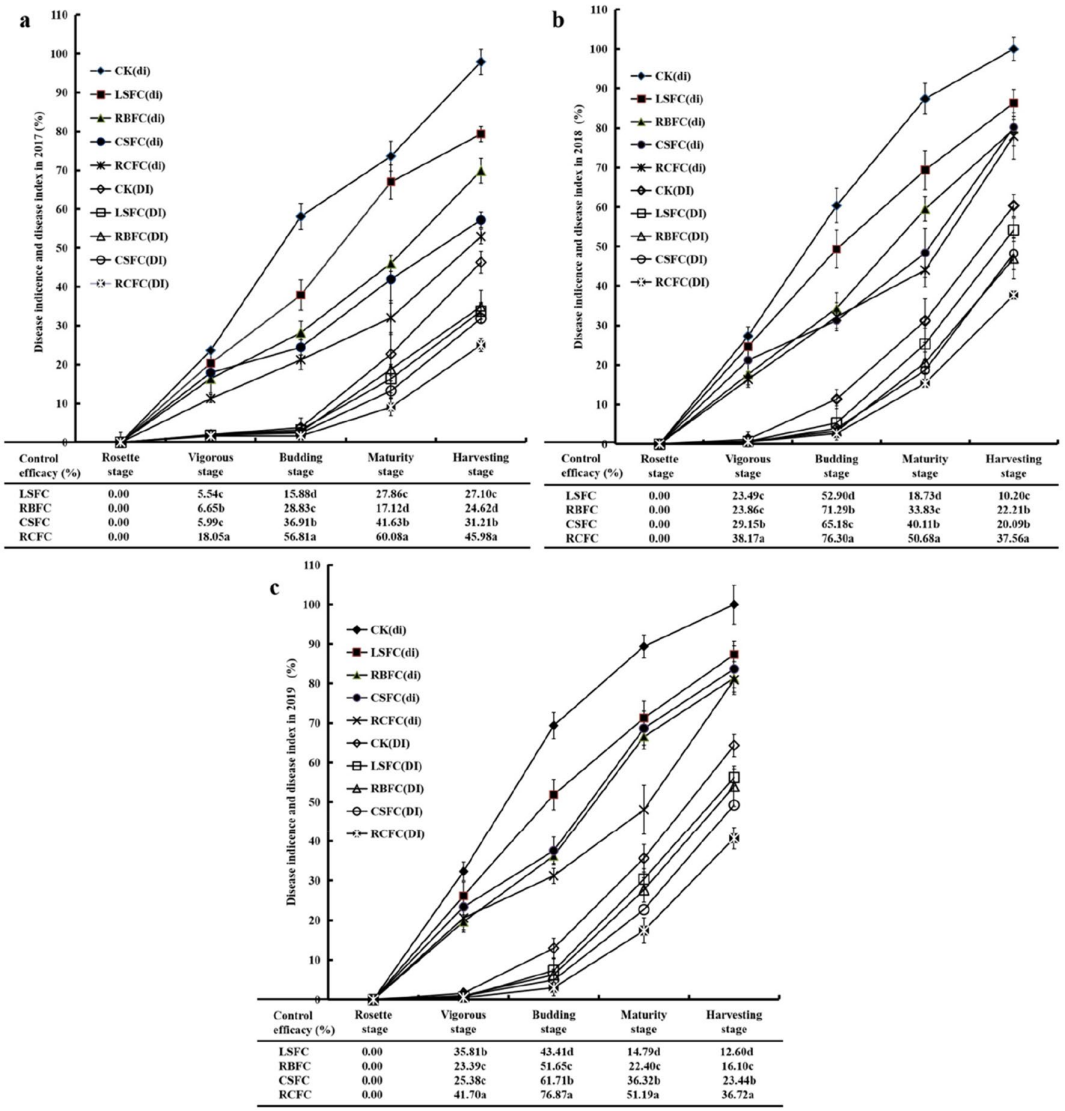
Disease incidence, disease index and control efficacy of five treatment groups at different stages in 2017 (**a**), 2018 (**b**) and 2019 (**c**). (The different letters in the same column indicate significant differences as determined by LSD test. *p* < 0.05).

Disease incidence and index with five treatments at different tobacco growth stages in different culture years were calculated. In 2017, the disease incidence and index of LSFC (the group with the application of alone liquid fermentation inoculants), RBFC (the group with the application of solid fermentation inoculants and rice bran), CSFC (the group with the application of solid fermentation inoculants and calcium cyanamide) and RCFC (the group with the application of solid fermentation inoculants, rice bran and calcium cyanamide) groups were lower than those of the CK group (the control group). Moreover, the disease incidence of RCFC group was the lowest in the five treatment groups. In 2018 and 2019, the variation tendency of disease incidence and index in CK, LSFC, RBFC, CSFC and RCFC groups was similar to that of 2017. Furthermore, the control efficacy was significantly higher in the RCFC group than in the LSFC, RBFC and CSFC groups at vigorous, budding, maturity and harvesting stages (Fig. 1).

### Physicochemical properties

**Figure 2:**
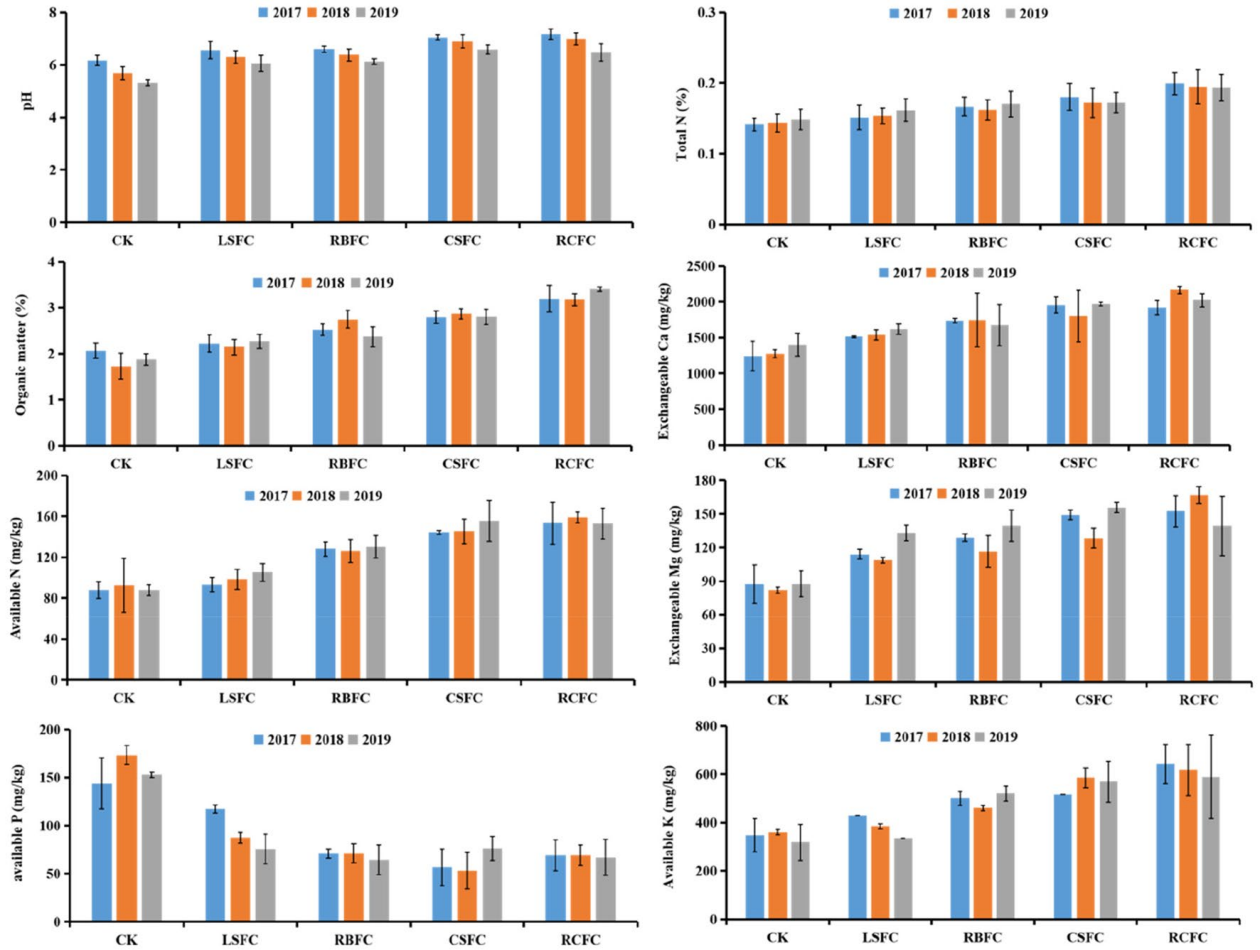
Soil physiochemical properties in different treatments from 2017 to 2019. Values are means of SD.

The physicochemical properties of different treatments were analyzed. The contents of organic matter (OM), alkaline nitrogen (alkaline N), total nitrogen (TN), available potassium (available K), exchangeable calcium (exchangeable Ca) and exchangeable magnesium (exchangeable Mg) in the CK, LSFC, RBFC, CSFC and RCFC groups increased successively, and the value of pH increased successively too. These soil physicochemical properties of the RCFC groups were slightly higher than other treatment groups. While, the contents of available P in the CK, LSFC, RBFC, CSFC and RCFC groups decreased successively (Fig. 2). In addition, the pH of different treatments showed decreased with the years from 2017 to 2019. What’s more, OM (Pearson =  − 0.713, *p* = 0.003), pH (Pearson =  − 0.745, *p* = 0.001), TN (Pearson =  − 0.524, *p* = 0.045), alkaline N (Pearson =  − 0.524, *p* = 0.045), available K (Pearson =  − 0.734, *p* = 0.002), exchangeable Ca (Pearson =  − 0.720, *p* = 0.002) and exchangeable Mg (Pearson =  − 0.758, *p* = 0.001) showed significantly negative correlation with the incidence of TBW (Table S1).

### Diversity analysis of soil bacterial community in the tobacco rhizosphere

**Figure 3:**
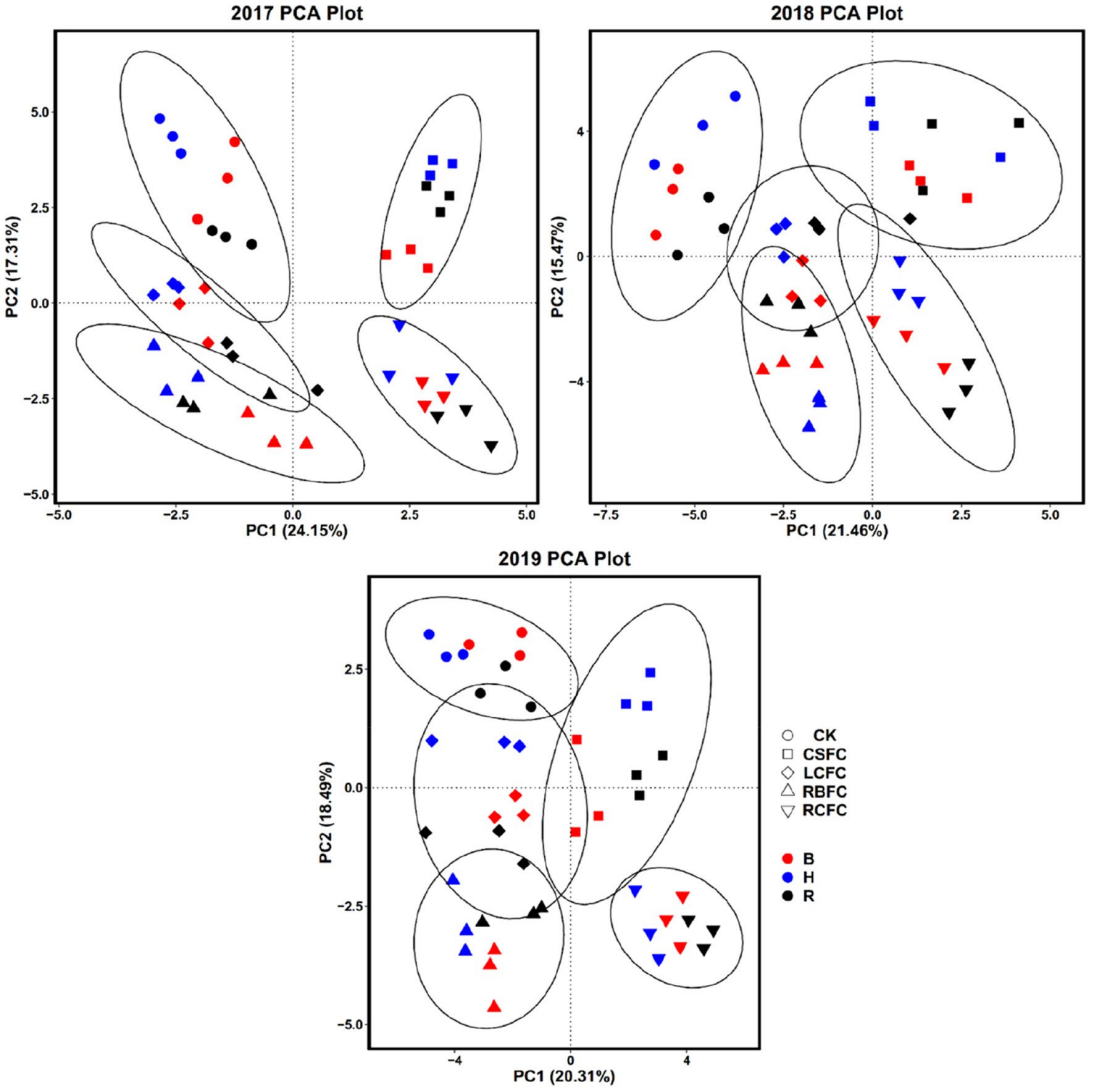
Two-dimensional plot of the principal components analysis (PCA) for the top ten abundant bacterial phyla in the different treatments at rosette (R), budding (B) and harvesting (H) stage in 2017, 2018 and 2019.

In total 712,691 high-quality raw sequences with the average length of 252 bps for bacteria were obtained from rhizosphere soil samples after quality filtering. The difference of OTUs, Sobs, Shannon, Simpson and Chao1 of bacteria richness and diversity were analyzed (Table S2). The number of OTUs in all samples ranged from 3852 to 6515, and the OTUs, Sobs and Chao 1 were higher in the rhizophere soil of the LSFC, RBFC, CSFC and RCFC groups than the CK group. Comparing with other treatment groups, the numbers of OTUs and Sobs of bacteria in the RCFC group was slightly higher. From 2017 to 2019, the rhizosphere bacterial community of different treatments changed with different trends at rosette, budding and harvesting stage. Pearson correlation showed the OTUs, Sobs, and Chao1 had significant negative correlation with the incidence of TBW (Table S3). Principal components analysis (PCA) were carried out using OTUs in the different treatments. In 2017, 41.46% of total variance was explained by the first two axes with 24.15% and 17.31% explanations in PC1 and PC2. In 2018, PC1 and PC2 explained 36.93% of total bacterial community. In 2019, 38.8% of total variance was explained by PC1 and PC2. From 2017 to 2019, the samples collected during different tobacco developmental stages within one treatment showed close distances, but distribution among different treatments was relatively discrete (Fig. 3), which indicated that the structure of bacterial community in the same sampling period was similar, and different treatments played important impact on the formation of bacterial community in the rhizosphere soil.

### The composition and structure of rhizospheric bacterial community in different treatments

The top ten abundant bacterial phyla were selected to compare the changes of bacterial community in rhizosphere soil of five treatment groups during tobacco growth period from 2017 to 2019 were *Proteobacteria*, *Acidobacteria*, *Actinobacteria*, *Chloroflexi*, *Gemmatimonadetes*, *Firmicutes*, *Bacteroidetes*, *Planctomycetes*, *Verrucomicrobia* and *Nitrospirate*. In 2017, the abundance of the phylum *Proteobacteria* included the pathogen *R. solanacearum* during harvesting stage in the CK, LSFC, RBFC, CSFC and RCFC groups were 58.15%, 56.33%, 43.91%, 44.60% and 37.61%, respectively. Furthermore, the abundance of *Proteobacteria* in the RCFC group was lower than that in the CK group. The abundance of the phylum *Firmicutes* included *B. amyloliquefaciens* ZM9 during harvesting stage in the CK, LSFC, RBFC, CSFC and RCFC groups were 2.02%, 2.65%, 3.07%, 5.28% and 6.53%, respectively (Fig. S1). The top ten abundant of bacterial phyla in five treatments changed irregularly during different tobacco growth stages from 2017 to 2019. For example, In the CK, the abundance of *Proteobacteria* was low at the rosette stage (R), increased at the budding stage (B), then decreased at the harvesting stage (H) from 2017 to 2019. In the CSFC and RCFC groups, the abundance of *Proteobacteria* were decreased at the budding stage (B), and increased at the harvesting stage (H). However, there are no regular changes in other treatment groups (Fig. S1). In addition, the top ten abundant phyla showed significantly negative correlation with the incidence of TBW, including *Chloroflexi* (Pearson =  − 0.627, *p* = 0.000), *Firmicutes* (Pearson =  − 0.455, *p* = 0.008), *Planctomycetes* (Pearson =  − 0.467, *p* = 0.006) and *Nitrospirae* (Pearson =  − 0.858, *p* = 0.000) (Table S4).

### Dynamic variations of *B. amyloliquefaciens* ZM9 and *R. solanacearum*

**Figure 4:**
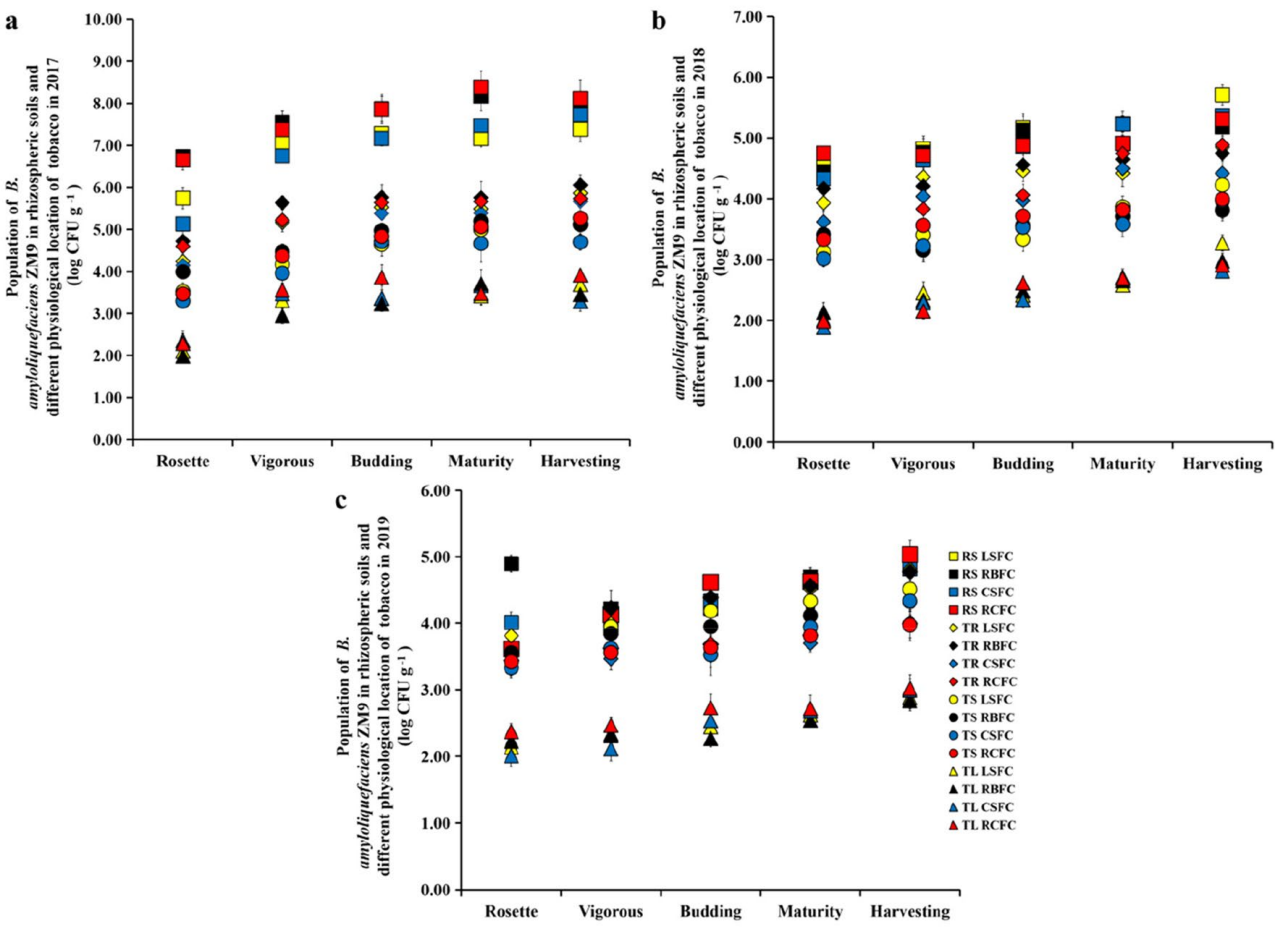
Population dynamic of *B. amyloliquefaciens* ZM9 in rhizospheric soil (RS), tobacco root (TR), stem (TS) and leaf (TL) of four treatment groups (LSFC, RBFC, CSFC and RCFC) at different tobacco growth stages in 2017 (**a**), 2018 (**b**) and 2019 (**c**).

**Figure 5:**
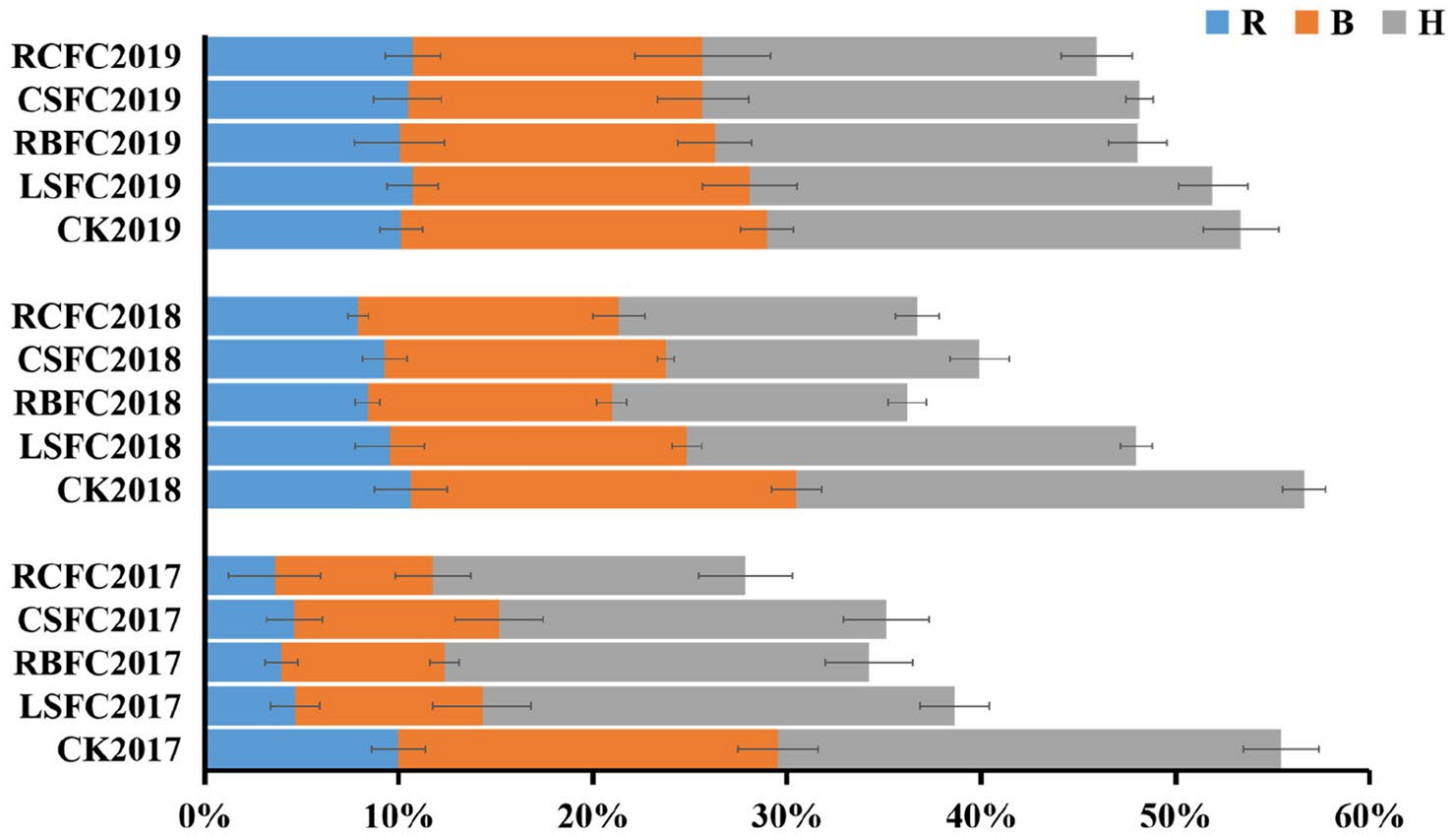
Population dynamics of the *R. solanacearum* in five treatment groups (CK, LSFC, RBFC, CSFC and RCFC) at rosette (R), budding (B) and harvesting (H) stage from 2017 to 2019.

The abundances of *B. amyloliquefaciens* ZM9 in rhizospheric soil, tobacco root, stem and leaf of four treatment groups (LSFC, RBFC, CSFC and RCFC) at different tobacco growth stages were calculated by the plated count method. The abundances of *B. amyloliquefaciens* ZM9 in rhizospheric soil were obviously higher than that in tobacco root, stem and leaf, and the abundances of *B. amyloliquefaciens* ZM9 in tobacco root, stem and leaf decreased successively. Additionally the abundances of *B. amyloliquefaciens* ZM9 in rhizospheric soil, tobacco root, stem and leaf of four treatment groups increased with the growth stages of tobacco (Fig. 4). From 2017 to 2019, the abundances of *B. amyloliquefaciens* ZM9 in rhizospheric soil decreased with years, and the difference in the abundances of *B. amyloliquefaciens* ZM9 in rhizospheric soil, tobacco root and stem gradually decreased with years (Fig. 3). As we focused on the TBW caused by *R. solanacearum*, we investigated the abundance of *R. solanacearum* in rhizospheric soil. In 2017, the abundance of *R. solanacearum* decreased significantly (*p* < 0.05) in the LSFC, RBFC, CSFC and RCFC groups, and the abundance of this pathogen in RCFC groups was the lowest among all treatment groups (Fig. 5). From 2017 to 2019, the abundance of *R. solanacearum* increased with the tobacco growth stages and years. Compared with the control group, the abundance of *R. solanacearum* in LSFC, RBFC, CSFC and RCFC groups decreased (Fig. 4). What’s more, the abundance of *R. solanacearum* showed significantly positive correlation with the incidence of TBW (*p* = 0.001). However, there was no significantly correlation between the abundance of *R. solanacearum* and bacterial community (Table S5). The linear regression analysis showed that the abundance of *R. solanacearum* was negatively correlated with the abundances of *B. amyloliquefaciens* ZM9 (Fig. S2).

### Relationships between bacterial community and soil physicochemical properties

**Figure 6:**
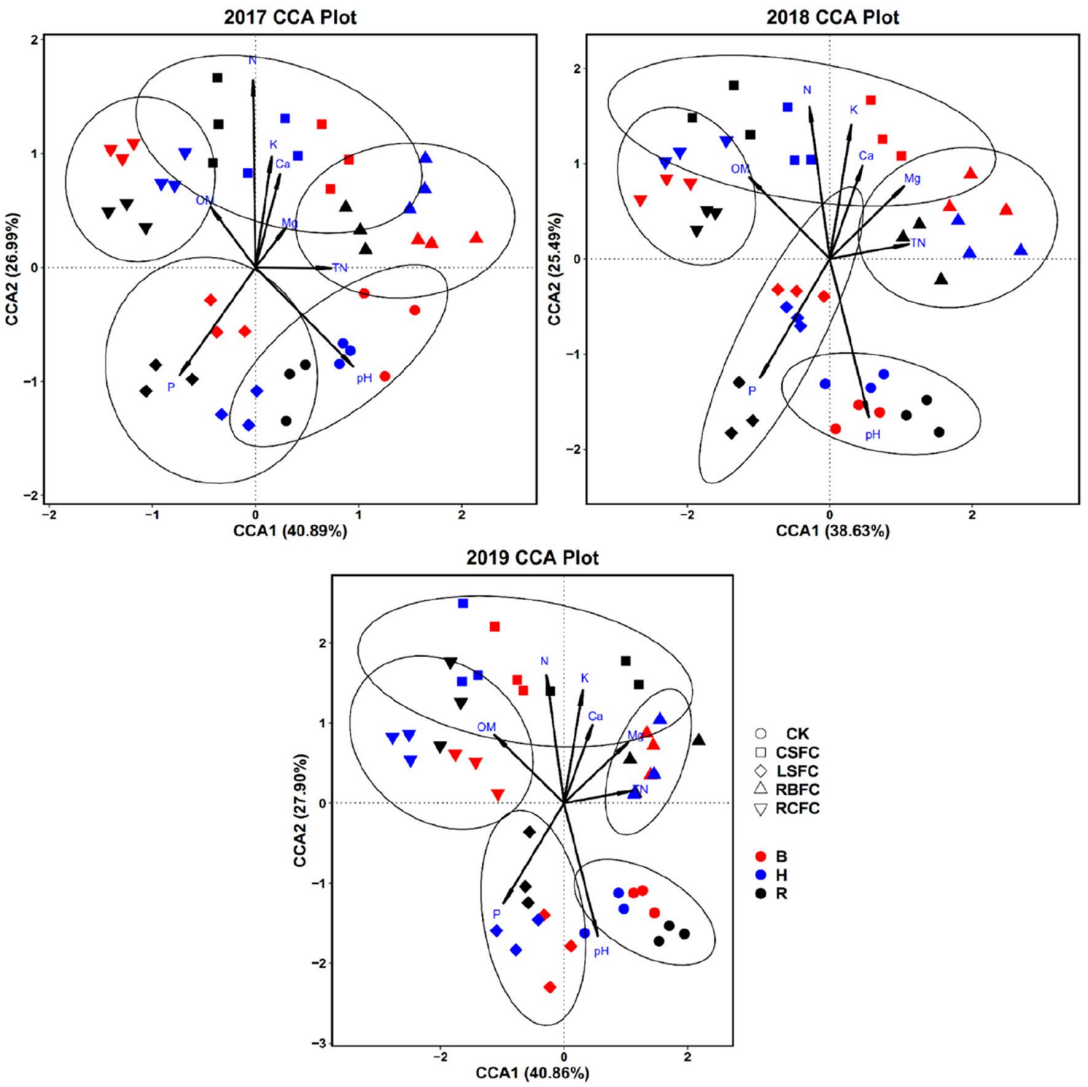
Canonical correspondence analysis (CCA) of the relationship between rhizospheric bacterial community and soil physicochemical properties in five treatment groups (CK, LSFC, RBFC, CSFC and RCFC) at rosette (R), budding (B) and harvesting (H) stage in 2017, 2018 and 2019. The soil properties are indicated with arrows, including soil pH, organic matter (OM), total nitrogen (TN), alkaline nitrogen (N), available phosphorous (P), available potassium (K), exchangeable calcium (Ca), exchangeable magnesium (Mg) content. The percentage of variation is explained by each axis.

The relationships between bacterial community structure and soil physicochemical properties were analysed with canonical correspondence analysis (CCA) from 2017 to 2019. Eight factors including pH, OM, alkaline N, TN, available K, available P, exchangeable Ca and exchangeable Mg were selected for CCA, and results showed that the treatments of CK, LSFC, RBFC, CSFC and RCFC were separated from each other (Fig. 6). These variables explained 67.88%, 64.12% and 68.76% of bacterial community variation in 2017, 2018 and 2019, respectively. The bacterial community composition in CK and LSFC groups were positively correlated with P and pH, but CSFC, RBFC and RCFC groups show negatively correlation with available P and pH. OM, available K, alkaline N, exchangeable Ca and exchangeable Mg positively correlated with CSFC, RBFC and RCFC groups, and negatively correlated with CK and LSFC groups (Fig. 6). The longer arrows of environmental factors showed that pH, OM, alkaline N, available K and available P play major roles in the formation of soil bacterial community structure, and pH, available P and available K best explained the differences between treatments, which was validated by Monte Carlo test (Table S6).

### Beneficial bacteria variation in response to different treatments

In this long-term field experiment, the relative abundance of beneficial bacteria in the tobacco rhizosphere in five treatment groups were compared. The relative abundance of beneficial bacteria in LSFC, RBFC and RCFC groups were significantly higher than that in the CK (Fig. S3). In 2017, among the beneficial bacteria in the tobacco rhizosphere, the relative abundance of *Acinetobacter*, *Azospirillum*, *Bradyrhizobium*, *Chthonomonas*, *Granulicella*, *Hyphomicrobium*, *Mesorhizobium*, *Pseudomonas*, *Psychrobacter* and *Stenotrophomons* in the LSFC, RBFC, CSFC and RCFC groups were higher than in the CK. Moreover, the relative abundance of *Acinetobacter*, *Bradyrhizobium*, *Pseudomonas* and *Psychrobacte*r increased significantly in the LSFC, RBFC, CSFC and RCFC groups, and these beneficial bacteria were slightly higher in RCFC (Fig. S3b). In 2018 and 2019, the variation tendency of the beneficial bacteria in different treatment groups was similar to that in 2017 (Fig. S3). What’s more, Pearson analysis indicated that *Acinetobacter*, *Azospirillum*, *Bradyrhizobium*, *Chthonomonas*, *Granulicella*, *Hyphomicrobium*, *Mesorhizobium*, *Pseudomonas*, *Psychrobacte*r and *Stenotrophomons* showed strong negative correlation with the incidence of TBW (Table S7).

### Antagonistic bacteria variation in response to different treatments

For further exploring the distribution of the antagonistic bacteria in different treatments, the abundances of antagonistic bacteria were calculated (Fig. S4). The relative abundance of antagonistic bacteria in LSFC, RBFC and RCFC groups were higher than in the CK. Furthermore, the relative abundance of *Actinospica*, *Bacillus*, *Burkholderia*, *Catenulispora* increased significant in the LSFC, RBFC, CSFC and RCFC groups (Fig. S4). These antagonistic bacteria had significant negative correlation with the incidence of TBW (Table S8). In addition, antagonistic bacteria in LSFC, RBFC and CSFC groups decreased with years from 2017 to 2019 (Fig. S4).

## Discussion

In previous investigation, *B. amyoliquefaciens* ZM9 as an efficient biocontrol agent could suppress TBW by producing lipopeptides to suppress *R. solanacearum* and regulating the tobacco rhizosphere microbial community in a field trial^5^. However, the long-term effects of *B. amyloliquefaciens* ZM9 with organic fertilizers or soil amendments on rhizopshere soil physicochemical properties, microbial community and the incidence of bacterial wilt is still unclear. In this study, *B. amyloliquefaciens* ZM9, rice bran and calcium cyanamide were applied to tobacco fields in different ways. Through long-term monitoring for three years, we found that rice bran and calcium cyanamide assitant with *B. amyloliquefaciens* ZM9 could effectively suppressed TBW through changing soil physicochemical properties and bacteria abundances.

Through three years of long-term monitoring, there was no significant change in the control efficacy of TBW by the combined application of *B. amyloliquefaciens* ZM9, rice bran and calcium cyanamide (Fig. 1). The result of present study revealed that the integrated biocontrol method is sustainable for the prevention and treatment of TBW. Since researchers indicated that the combined application of antagonistic bacteria and organic fertilizer or soil amendments can be effective in the prevention and control of various soil-borne diseases^15,18^. In this study, a significantly negative relationship between some soil properties (OM, alkaline N, available K, exchangeable Ca, exchangeable Mg and pH) and the incidence of TBW was well documented (Table S1). We infer that the integrated biocontrol measure may reduce the incidence of TBW by regulating soil physicochemical properties. Calcium cyanamide can increase soil pH, total N, alkaline N and exchangeable Ca^22^. Additionally, rice bran provides soil with C, N, P and other nutrients^21^. C may increase the soil OM, and OM improves soil fertility and crop yields^23^. At the same time, OM is an important source of microbial nutrients^24^. Increase pH is important for inhibited the survival of *R. Solanacearum*, increase N and P meet the need of plant growth^20,23^. However, after 3 years of long-term monitoring, the pH values of different treatments decreased with the years (Fig. 2), which consistently with previous reports^6,25^. That may attribute to the accumulation of allelochemicals. Moreover, the soil physicochemical properties were related to the microbial community. As reported, pH may stress microbial cells to select specific microbial groups and affect the composition of soil microbial community^26^. P application can cause an increase in bacterial diversity, with increase *Actinobacteria* and decreased *Acidobacteria* in soils^27^. In this study, CCA results also showed that soil physicochemical properties relevant to the bacterial community structure (Fig. 6). Here, the available P in the treatment groups were decreased compared with control group (Fig. 2). It maybe the treatment groups plants absorb much more effective P, and the abundance of microorganisms were higher in the treatment groups than the control group, these further promoted the treatment groups consumption of P.

The incidence of TBW was also associated with soil microbial community diversity, as indicated by OTUs, Sobs and Chao1. Bacterial OTUs, Sobs and Chao1 were more diverse in biocontrol treatment groups than in the control group (Table S2). Additionally, OTUs and Sobs had significant negative correlation with the incidence of TBW (Table S3). This is similar to previous report that the highly rhizosphere soil microbial community diversity would decrease the incidence of TBW^28^. Shen et al.^29^ found that organic fertilizer could increase soil microbial activity, enhance competition for nutrients for pathogens. A recent report indicated that soil amendment calcium cyanamide could supply sufficient N, and increase soil pH, microbial diversity^30^. Our findings also demonstrated that both the alone application rice bran, calcium cyanamide or *B. amyoliquefaciens* ZM9 and the combined application of rice bran, calcium cyanamide and *B. amyoliquefaciens* ZM9 (RCFC group) could effect on the variation of bacterial community, while the combined application would bring higher bacteria community diversity than the other treatments. We also hypothesized that the rhizospheric bacterial community varies at different plant growth stages. The most abundant phyla bacterial detected in this study were *Proteobacteria*, *Acidobacteria*, *Actinobacteria* and *Chloroflexi* (Fig. S1), supporting the findings of others^4,31,32^. *Proteobacteria* paly important roles in C, N and S cycles^23,33^. *Actinobacteria* as the third most prominent was negative correlation with the incidence of TBW. Liu et al.^34^ revealed that antgonistic bacteria *Actinobacteria* can produce a diverse range of antibiotics and induce plant resistance to control plant bacterial diseases. These bacterial can rapidly respond to environmental perturbations leading to dynamic changes in abundance, activity and composition^35^. It is noted that these rhizospheric bacterial changed with different trends during different tobacco growth stages from 2017 to 2019 (Fig. S1).

In this study, we have successfully isolated *B. amyoliquefaciens* ZM9 through its ability to colonize the tobacco root, stem, leaf, and rhizosphere soil, and the results were consistent with our previous findings^5^. This indicated that *B. amyloliquefaciens* ZM9 had colonization ability. Also, we found that the abundances of *B. amyloliquefaciens* ZM9 in rhizospheric soil gradually decreased under continuous cropping (Fig. 4). We suspected that the reason why colonization of *B. amyloliquefaciens* ZM9 strain was decreased with years from 2017 to 2019 was that the concentration of *B. amyloliquefaciens* ZM9 in the medium was decreased continuously with the years. Linear fitting showed that the abundance of *R. solanacearum* was negatively correlated with the abundances of *B. amyloliquefaciens* ZM9 (Fig. S2). Corresponding with reality, the decreased abundance of *B. amyloliquefaciens* ZM9 can lead the increased *R. solanacearum*, because the antagonistic effect will become weaker and weaker, and this also proves the antagonistic effect of *B. amyloliquefaciens* ZM9 on *R. solanacearum* from another aspect.

In the current study, during three years of long-term monitoring, the variation tendency of the beneficial bacteria in different treatment groups was similar, and many beneficial bacteria involved in element cycling and promoted plant growth. Some beneficial bacteria in the rhizosphere soil were higher abundances in the LSFC, RBFC, CSFC and RCFC groups than in the CK, including *Acinetobacter*, *Azospirillum*, *Bradyrhizobium*, *Mesorhizobium*, *Pseudomonas*, *Psychrobacter* and *Stenotrophomons* (Fig. S3). This indicated that biocontrol inoculants might stimulate beneficial bacteria. Some of these beneficial bacteria can produce hormones to stimulate plant growth, increase plant resistance to stress^5^. While, *Mesorhizobium* can assist plant to survive by producing biofilm^36^. Moreover, some species of *Granulicella* were found to use different substances as carbon sources, and several species of *Chthonomonas* have a special effect on the transition of organic carbon in the rhizosphere soil^37,38^. The bacteria *Hyphomicrobium* involved in nitrogen cycling and can perform partial or complete denitrification^39^. We also found that these beneficial bacteria showed strong negative correlation with the incidence of TBW. These beneficial bacteria can directly and effectively control TBW by competing for nutrients, space, and inducing systemic resistance^40,41^.

It has been well documented that *Actinospica*, *Bacillus*, *Burkholderia*, *Catenulispora*, *Chryseobacterium*, *Clostridium*, *Flavobacterium*, *Haliangium*, *Microbispora*, *Paenibacillus*, *Rhodopseudomonas*, *Sphingomonas*, *Staphyloccus*, *Stenotrophomonas* and *Streptomyces* as antagonistic bacteria can mitigate many soil-borne diseases^42–47^. Antagonistic bacteria *Actinospica*, *Catenulispora*, *Haliangium*, *Microbispora* can form a alliance of antibiotic producers and change bacterial community structure^35^. In addition, there were several endophytic or rhizobacteria such as *Burkholderia*, *Sphingomonas*, *Staphyloccus*, *Bacillus* and *Rhodopseudomonas* can promote plant growth and inhibit pathogenic bacteria^44,48–51^. These antagonistic bacteria were higher abundances in LSFC, RBFC and RCFC groups than in the CK (Fig. S4). Moreover, these antagonistic bacteria were negatively related to the incidence of TBW (Table S8), revealing that biocontrol teatment groups may improve the growth of antagonistic bacteria and contribute to pathogen inactivation. We also found antagonistic bacteria in LSFC, RBFC and CSFC groups decreased with years from 2017 to 2019 (Fig. S4), indicating the antagonistic bacteria decreased under continuous cropping.

## Conclusions

The combined application of rice bran and calcium cyanamide could promote the colonization ability of *B. amyoliquefaciens* ZM9 and effectively suppress TBW by regulating soil physicochemical properties, and enriching the beneficial bacteria and antagonistic bacteria in the soils. Our results provide a promising strategy for TBW control by adding soil amendment calcium cyanamide and organic fertilizer rice bran.

## Materials and methods

### Field experiment

Field experiment were performed in a 15 years history of continuous cropping tobacco field in Xuan’en County (109°26ʹ20ʺ E, 29°59 ʹ 55 ʺ N), Enshi City, Hubei province, China from April to September in 2017, 2018 and 2019. The incidence of TBW in this field had been above 95% every year for the past five years before this study. Tobacco seedings (Yunyan87) were grown according to our previous study^5^. *B. amyloliquefaciens* ZM9 (Genbank: KF906355.1) was used as the antagonistic bacteria in this study. Five treatments were established: (1) the control group (CK, without any pesticide); (2) the group with the application of alone liquid fermentation inoculants (LSFC): *B. amyloliquefaciens* ZM9 was incubated and irrigated into the tobacco root as previous study^5,21^; (3) the group with the application of solid fermentation inoculants and rice bran (RBFC): 300 g of rice bran was mixed with the 100 mL of the diluted *B. amyloliquefaciens* ZM9 culture (1.0 × 10^7^ CFU/mL) and fermented for 3 days, adjusted to a final concentration to 2.5 × 10^6^ CFU/g, and then 40 g was applied to soil for each plant when the tobacco was transported; (4) the group with the application of solid fermentation inoculants and calcium cyanamide (CSFC): 150 kg/hm^2^ of calcium cyanamide were applied during soil preparation process, and then irrigated the diluted *B. amyloliquefaciens* ZM9 culture into the tobacco roots; and (5) the group with the application of solid fermentation inoculants, rice bran and calcium cyanamide (RCFC): 150 kg/hm^2^ of calcium cyanamide were applied during soil preparation process, and then loaded same amount of *B. amyloliquefaciens* ZM9 as described in LSFC. All treatments were performed only in the first year and then monitored 3 years continuously. There were 180 tobacco plants per treatment, three replicates (60 plants in each replicate). All treatments and replicates were randomly placed in the field, and groups were irrigated with the same amount of initial water.

### Disease incidence, index and control efficacy calculation

The TBW disease index (DI) based on severity scale of 0–9 was described in a previous study^23^. Briefly, “0” represents the plants without visible symptoms; “1” represents the presence of occasional chlorotic spots on stems, or less than half of the leaves wilted on unilateral stems; “3” represents the presence of a black streak less than half the height of the stem, or between half to two-thirds of the leaves wilted on unilateral stems; “5” represents the presence of a black streak over half the length of the stem, but not reaching the top of the stem, or more than two-thirds of the leaves wilted on unilateral stems; “7” represents the presence of a black streak reaching the top of the stem, or all leaves wilted; and “9” represents the dead plant. Based on the number of plants in each rating scale, disease incidence (di) and disease index (DI) of TBW were calculated as di = n′/ N × 100% and DI = ∑(r × n)/(N × 9) × 100. where n′ is the total number of infected tobacco plants, r is the rating scale of disease severity, n is the number of infected tobacco plants with a rating of r, and N is the total number of plants. Control efficacy = [(di of control − di of treatment)/di of control] × 100%. The di, DI and control efficacy were measured at 1 week, 3 weeks, 6 weeks, 9 weeks and 15 weeks after irrigation in 2017, 2018 and 2019, respectively.

### Rhizosphere soil sampling and physicochemical properties analysis

The rhizosphere soil was collected by five-spot-sampling method from 2017 to 2019, respectively.$\sum (\mathrm{r}\times \mathrm{n})/(\mathrm{N}\times 9)\times 100$ $\sum (\mathrm{r}\times \mathrm{n})/(\mathrm{N}\times 9)\times 100$ Then transported to the laboratory, stored at − 80 °C for microbiological analysis, and at room temperature for physicochemical properties analysis. Soil samples were air-dried and ground (< 2 mm) to determine available nutrients and soil characteristics. pH was determined by potentiometric method, organic matter (OM) was determined by potassium dichromate volumetric method, total nitrogen (TN) was determined by Kjeldahl method, alkaline nitrogen (alkaline N) was determined by alkali solution diffusion method, available phosphorus (available P) was determined by the sodium bicarbonate method, available potassium (available K) was determined by flame spectrophotometer, and exchangeable calcium (exchangeable Ca), exchangeable magnesium (exchangeable Mg) was determined by atomic absorption spectrophotometry^24^.

### DNA extraction

Soil DNA was extracted from 0.5 g rhizosphere soil using the FastDNA Spin Kit (MP NA gene. The V4 region of 16S rRNA Biomedicals, USA) following the manufacture’s protocol. The integrity of DNA samples were determined by 1% agarose gel electrophoresis. Then the concentration and purity of the DNA were determined using a Nanodrop ND-1000 Spectrophotometer (Nanodrop Technologies, Wilmington, DE, USA).

### DNA sequence data collection and analysis

The extracted soil DNA was used as template to amplify 16S rRNA gene were amplified with the primers 515F (5′-GTGCCAGCMGCCGCGGTAA-3′) and 806R (5′-GGACTACHVGGGTWTCTAAT-3′)^5^. All PCR reactions were performed on Illumina HiSeq platforms (Illumina Inc., USA) at Novogene Bioinformatics Technology Co., Ltd (Beijing, China). The library quality was assessed on the Qubit@ 2.0 Fluorometer (Termo Scientifc) and Agilent Bioanalyzer 2100 system. Sequences analysis was performed by Uparse software (Version 7.0.1001, http://drive5.com/uparse/). Sequences with ≥ 97% similarity were assigned to the same OTUs. Each representative sequence was screened for further taxonomic information annotation. FASTX Toolkit 0.0.13 software package was used to preliminarily filtrate the raw sequence data with removing the low mass base at the tail of the sequence (Q value less than 20) and the sequences with lengths less than 35 bp. The sequence quality was statistically analyzed by CASAVA1.8, and finally, the length of the valid reads was approximately 250 bp. The operational taxonomic units (OTUs), observed-species (Sobs), Shannon, Simpson and Chao1 were calculated to evaluate richness and diversity of soil microbial community.

### Dynamic variation of *B. amyloliquefaciens* ZM9 and *R. solanacearum* in field experiments

Tobacco plants were uprooted from four treatment groups (LSFC, RBFC, CSFC and RCFC) at different growth stages to evaluate the population dynamic of *B. amyloliquefaciens* ZM9 in the rhizosphere soil (RS), tobacco root (TR), stem (TS) and leaf (TL) by the plate count method with three biological replicates^5^ (Fig. S5). The media used in this study for *B. amyloliquefaciens* ZM9 plate count was the LB selective media with 200 mg/mL rifampin. The abundances of *R. solanacearum* in rhizospheric soil of five treatment groups (CK, LSFC, RBFC, CSFC and RCFC) at different tobacco growth stages were calculated based on 16S rRNA gene sequencing data.

### Statistical analysis

The data were analyzed with Microsoft Excel 2007 and SPSS version 18.0 (IBM, USA). Differences between treatments were assessed by one-way analysis of variance (ANOVA) and least significant difference (LSD) test (*p* < 0.05). Correlation analysis was conducted by Pearson (2- tailed). Principal components analyzed (PCA) with the weighted UniFrac distance and canonical correspondence analysis (CCA) were carried out using the vegan package in R (Version 2.15.3). Monte Carlo test were used to construct dissimilarity matrices of bacterial community and soil physicochemical properties.

## Supplementary Information

Supplementary Information.
