# Supplementary material for: Effects of integrated biocontrol on bacterial wilt and rhizosphere bacterial community of tobacco
Source: Sci Rep. 2021 Jan 29;11:2653. doi: 10.1038/s41598-021-82060-3 (PMC7846572; doi:10.1038/s41598-021-82060-3)
Supplement: Supplementary file 1 — Supplementary Information. [file 41598_2021_82060_MOESM1_ESM.docx]

Supporting information

**Effects of integrated biocontrol on bacterial wilt and rhizosphere bacterial community of tobacco**

**Yun Hu^1a^, Yanyan Li^2a^, Xiaoqiong Yang^1^, Chunli Li^1^, Lin Wang^3^, Ji Feng^2^, Shouwen Chen^1^, Xihong Li^2*^ & Yong Yang^1*^**

^1^State Key Laboratory of Biocatalysis and Enzyme Engineering, School of life science, Hubei University, Wuhan 430062, China.

^2^Tobacco Research Institute of Hubei Province, Wuhan 430030, China.

^3^Hubei Tobacco Industry Co., Ltd., Wuhan 430040, China.

*E-mails: [yangyong@hubu.edu.cn,](mailto:yangyong@hubu.edu.cn,) [lxh885@126.com](mailto:lxh885@126.com).

^a^Authors contribute equally to present work.


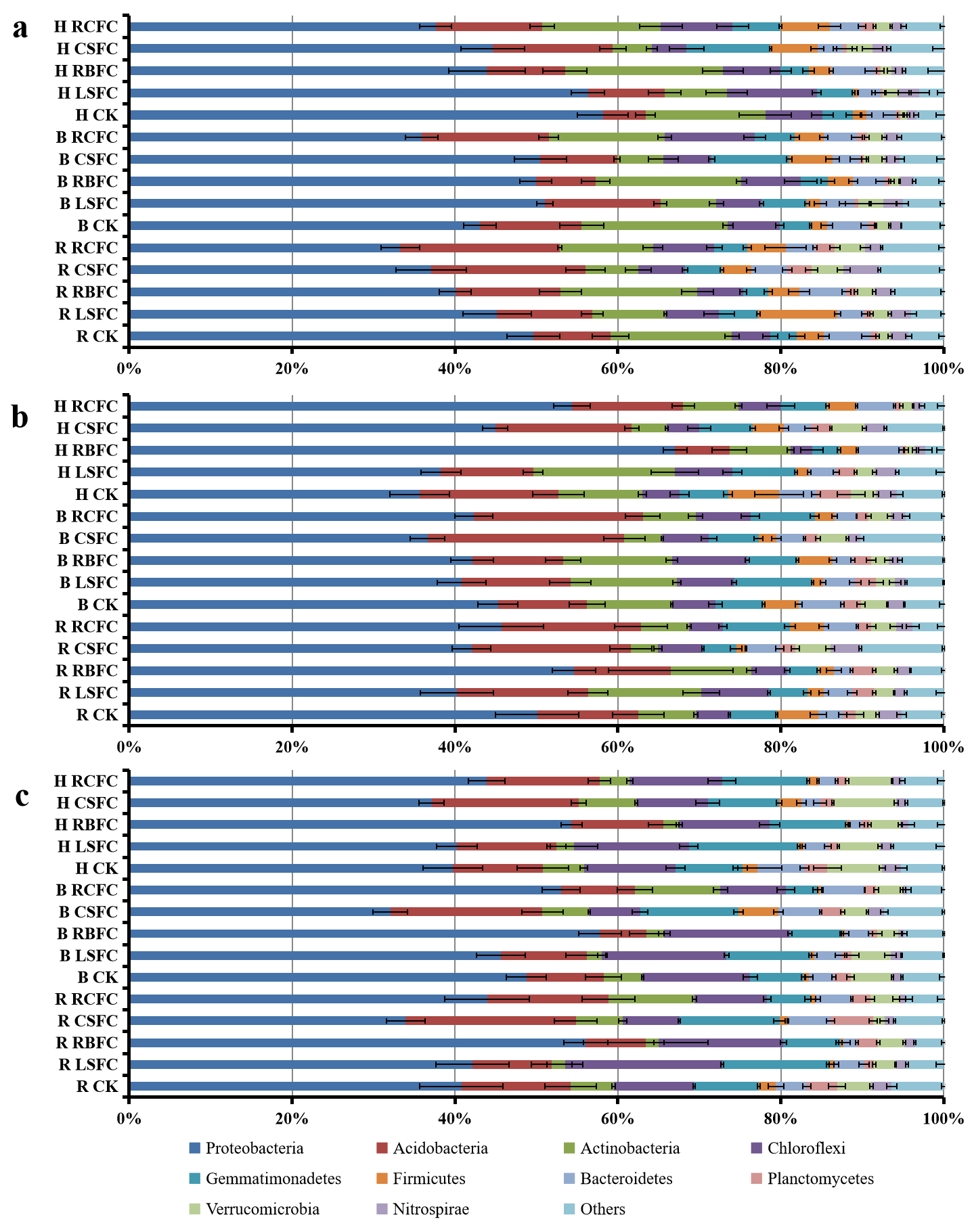


**Figure S1.** Comparison of top ten abundant phyla of bacterial community in rhizospheric soil of five treatment groups (CK, LSFC, RBFC, CSFC and RCFC) at rosette (R), budding (B) and harvesting (H) stage in 2017 (a), 2018 (b) and 2019 (c).

**
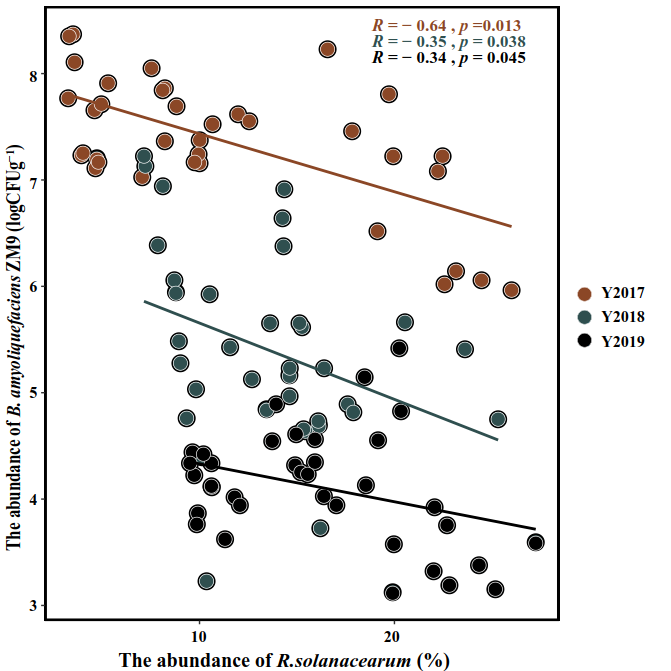
**

**Figure S2.** The relationship between the abundance of *amyoliquefaciens ZM9* and the abundance of *R. solanacearum* from 2017 to 2019.

**
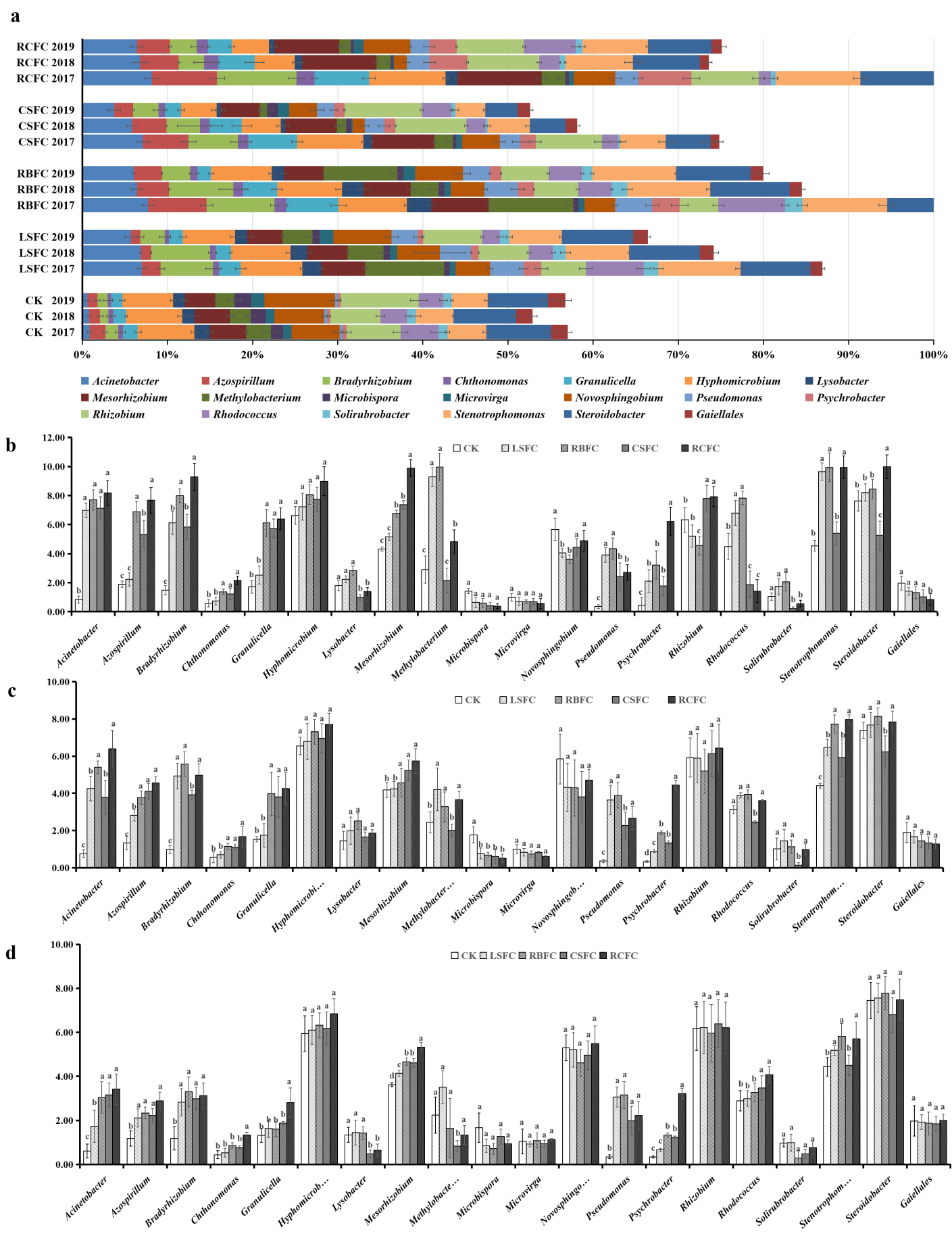
**

**Figure S3.** Comparison of relative abundance of beneficial bacteria in five treatment groups (CK, LSFC, RBFC, CSFC and RCFC) from 2017 to 2019 (a). The relative abundance of beneficial bacteria in different treatment groups in 2017 (b), 2018 (c) and 2019 (d). Values are means of SD. Bars with different letters are significantly different at *p* < 0.05 by LSD test. .


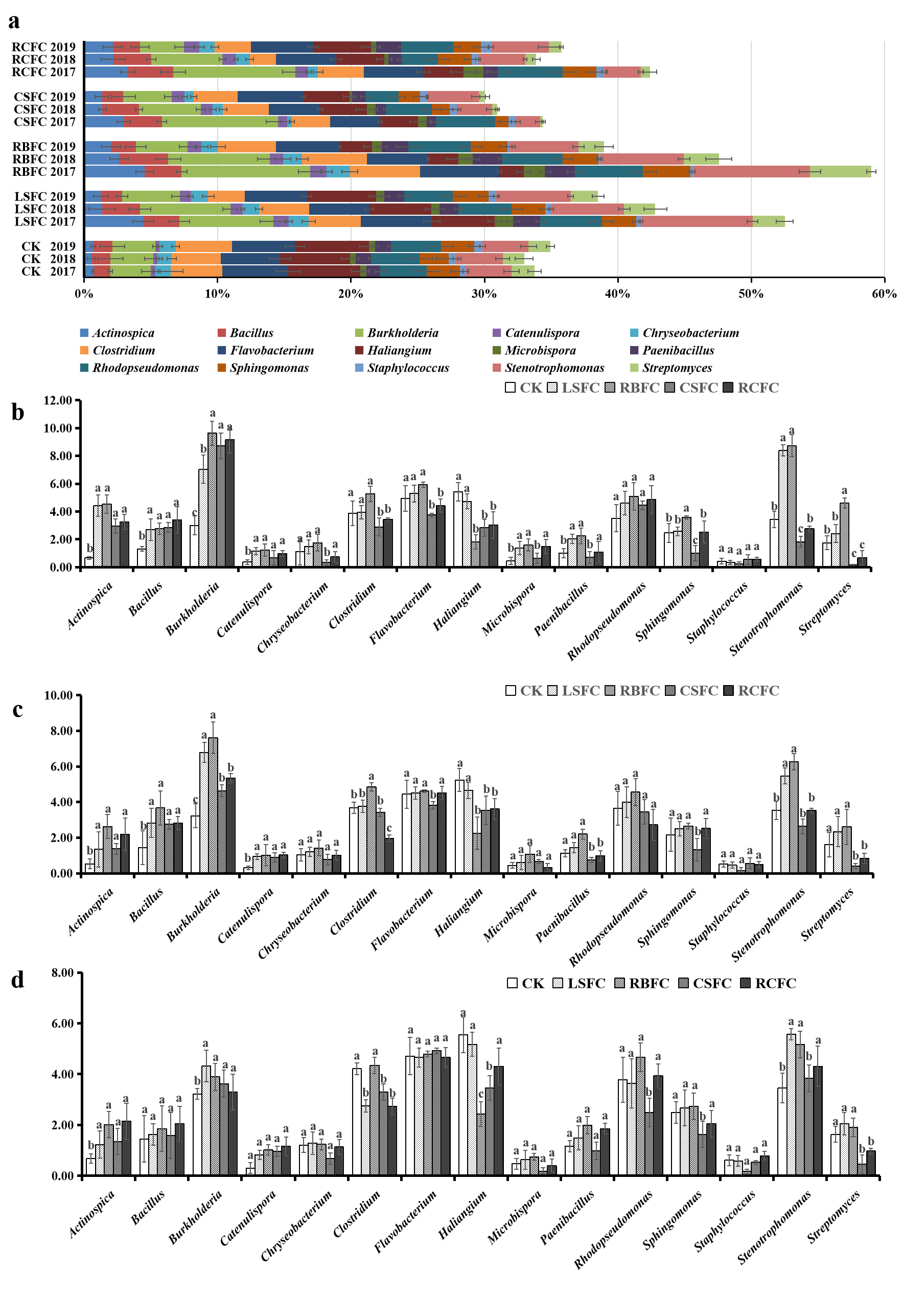


**Figure S4.** Comparison of relative abundance of antagonistic bacteria in five treatment groups (CK, LSFC, RBFC, CSFC and RCFC) from 2017 to 2019 (a). The relative abundance of antagonistic bacteria in different treatment groups in 2017 (b), 2018 (c) and 2019 (d). Values are means of SD. Bars with different letters are significantly different at *p* < 0.05 by LSD test.

**
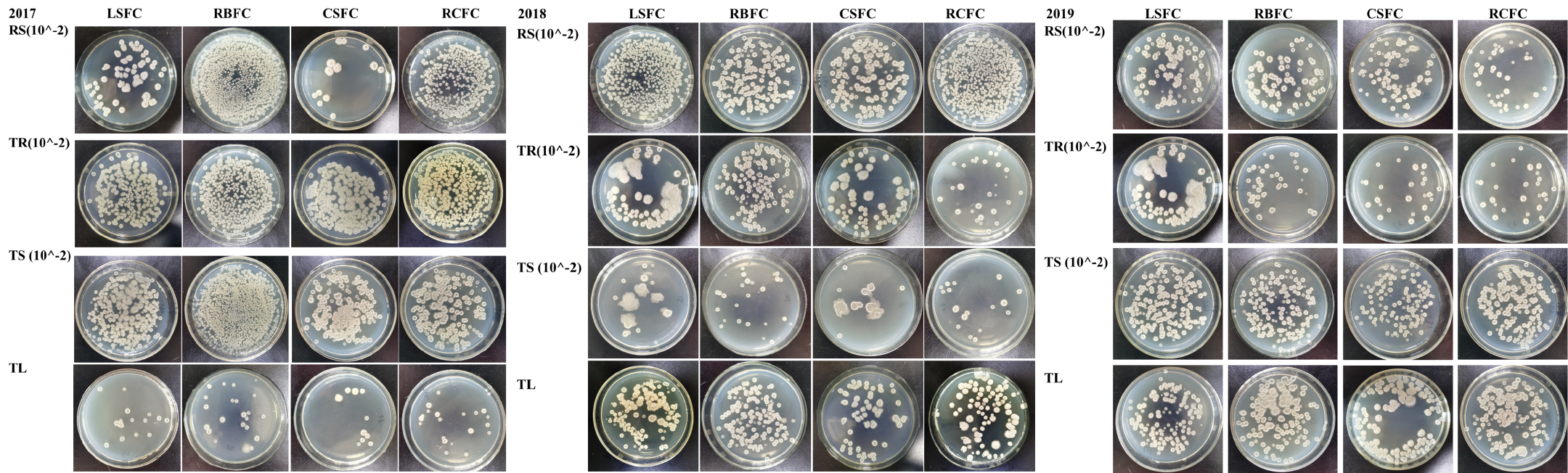
Figure S5.** Plate count figure of *B. amyloliquefaciens* ZM9 in rhizospheric soil (RS), tobacco root (TR), stem (TS) and leaf (TL) of four treatment groups (LSFC, RBFC, CSFC and RCFC) at rosette stage.

**Table S1.** Pearson correlation analysis between disease incidence of tobacco bacterial wilt and soil physicochemical properties. (* and ** indicate correlation is significant at *p* < 0.05 and *p* < 0.01.**)**

|  | **pH** | **organic matter** | **total N** | **alkaline N** | **available P** | **available K** | **exchangeable Ca** | **exchangeable Mg** |
| --- | --- | --- | --- | --- | --- | --- | --- | --- |
| **Pearson** | −0.745** | −0.713** | −0.524* | −0.698** | 0.729** | −0.734** | −0.720** | −0.758** |
| ***p* value** | 0.001 | 0.003 | 0.045 | 0.004 | 0.002 | 0.002 | 0.002 | 0.001 |

**Table S2.** Alpha diversity index of bacteria in different treatments. (Values are means ± standard deviation. The different letters in the same column indicate significant differences as determined by LSD test. *p* < 0.05.)

| **Year** | **Growth stage** | **Treatment** | **OTUs** | **observed_species**  **(Sobs)** | **Shannon index** | **Simpson index** | **Chao1 index** |
| --- | --- | --- | --- | --- | --- | --- | --- |
| **2017** | **Rosette** | CK | 4132.00 ± 347.33b | 3332.34 ± 310.6a | 9.21 ± 0.06a | 0.99 ± 0.01a | 4280.25 ± 235.50a |
|  |  | LSFC | 4988.21 ± 149.13a | 3683.33 ± 365.88a | 9.46 ± 0.25a | 0.95 ± 0.01a | 4910.21 ± 174.34a |
|  |  | RBFC | 5025.45 ± 262.81a | 3516.00 ± 477.68a | 9.30 ± 0.75a | 0.91 ± 0.01a | 4303.28 ± 498.27a |
|  |  | CSFC | 5274.31 ± 374.23a | 3576.33 ± 452.75a | 9.35 ± 0.06a | 0.93 ± 0.01a | 5052.82 ± 797.93a |
|  |  | RCFC | 5792.56 ± 461.11a | 3610.00 ± 667.49a | 9.53 ± 0.44a | 0.91 ± 0.01a | 4853.15 ± 1033.76a |
|  | **Budding** | CK | 3852.63 ± 232.89b | 3416.33 ± 94.30b | 9.24 ± 0.32a | 0.99 ± 0.01a | 4636.62 ± 534.82b |
|  |  | LSFC | 4333.23 ± 448.15b | 4054.67 ± 579.94a | 9.77 ± 0.21a | 0.96 ± 0.01a | 6319.48 ± 476.16a |
|  |  | RBFC | 5364.42 ± 244.45a | 4037.00 ± 531.79a | 9.86 ± 0.03a | 0.94 ± 0.02a | 4762.12 ± 633.96b |
|  |  | CSFC | 5522.36 ± 408.89a | 4127.00 ± 235.42a | 9.76 ± 0.09a | 0.95 ± 0.01a | 5567.09 ± 179.71a |
|  |  | RCFC | 5889.56 ± 133.46a | 4553.56 ± 106.01a | 9.10 ± 0.67a | 0.96 ± 0.03a  . | 5123.62 ± 484.64b |
|  | **Harvesting** | CK | 4287.27 ± 130.79c | 3734.33 ± 334.34c | 9.20 ± 0.10a | 0.97 ± 0.01a | 4826.96 ± 772.78b |
|  |  | LSFC | 4625.53 ± 213.78bc | 4752.33 ± 285.51a | 9.93 ± 0.09a | 0.96 ± 0.01a | 5931.16 ± 655.64a |
|  |  | RBFC | 5356.51 ± 496.67b | 4216.00 ± 56.82b | 9.56 ± 0.28a | 0.94 ± 0.05a | 5589.54 ± 461.86a |
|  |  | CSFC | 5061.42 ± 291.56b | 4536.12 ± 174.31a | 9.44 ± 0.31a | 0.96 ± 0.02a | 5384.66 ± 618.10a |
|  |  | RCFC | 6276.56 ± 171.78a | 4418.35 ± 324.69a | 9.51 ± 0.29a | 0.94 ± 0.02a | 5232.51 ± 279.25a |
| **2018** | **Rosette** | CK | 4052.13 ± 601.45b | 3214.11 ± 635.70b | 7.53 ± 2.05b | 0.98 ± 0.10a | 4283.72 ± 930.69b |
|  |  | LSFC | 5324.45 ± 388.26a | 4387.67 ± 308.89a | 10.26 ± 0.11a | 0.96 ± 0.01a | 5656.82 ± 796.63a |
|  |  | RBFC | 5385.46 ± 287.56a | 4429.16 ± 146.27a | 9.76 ± 0.62a | 0.96 ± 0.10a | 5048.78 ± 1204.53a |
|  |  | CSFC | 5379.45 ± 84.45a | 4339.67 ± 80.53a | 9.93 ± 0.08a | 0.95 ± 0.01a | 5981.79 ± 204.68a |
|  |  | RCFC | 5858.45 ± 462.56a | 4438.33 ± 194.51a | 9.45 ± 0.42a | 0.93 ± 0.01a | 5268.68 ± 416.39a |
|  | **Budding** | CK | 4761.36 ± 79.11b | 3575.42 ± 160.99b | 9.42 ± 0.28a | 0.99 ± 0.02a | 4528.76 ± 246.76b |
|  |  | LSFC | 5429.56 ± 312.42a | 4314.67 ± 189.29a | 10.18 ± 0.09a | 0.98 ± 0.01a | 5601.08 ± 537.20a |
|  |  | RBFC | 5326.76 ± 143.42a | 4259.76 ± 320.04a | 9.50 ± 0.12b | 0.97 ± 0.01a | 4654.35 ± 216.37b |
|  |  | CSFC | 5596.12 ± 234.56a | 4564.33 ± 341.59a | 9.44 ± 0.21b | 0.95 ± 0.01a | 5666.51 ± 62.01a |
|  |  | RCFC | 5699.89 ± 516.42a | 4494.67 ± 431.77a | 9.64 ± 0.29b | 0.95 ± 0.01a | 4944.93 ± 398.58b |
|  | **Harvesting** | CK | 4726.75 ± 511.78b | 3960.67 ± 727.13b | 9.01 ± 2.02a | 0.97 ± 0.02a | 4346.42 ± 327.13a |
|  |  | LSFC | 5339.23 ± 503.12a | 4310.33 ± 356.03a | 10.27 ± 0.11a | 0.99 ± 0.01a | 5334.93 ± 842.29a |
|  |  | RBFC | 5544.67 ± 292.23a | 4371.45 ± 323.03a | 9.60 ± 0.41a | 0.96 ± 0.07a | 4601.07 ± 840.79b |
|  |  | CSFC | 5392.76 ± 411.23a | 4498.67 ± 92.99a | 10.15 ± 0.15a | 0.95 ± 0.07a | 5241.39 ± 813.61a |
|  |  | RCFC | 5702.65 ± 352.45a | 4486.67 ± 416.31a | 9.59 ± 0.35a | 0.98 ± 0.03a | 5436.91 ± 326.40a |
| **2019** | **Rosette** | CK | 4832.61 ± 70.42d | 3454.67 ± 264.37b | 9.21 ± 0.06a | 0.98 ± 0.01a | 3429.70 ± 292.92c |
|  |  | LSFC | 5257.42 ± 117.45c | 4296.00 ± 898.55a | 9.32 ± 0.35a | 0.98 ± 0.01a | 4544.29 ± 180.84b |
|  |  | RBFC | 5517.24 ± 80.21b | 4246.00 ± 63.59a | 10.00 ± 0.11a | 0.93 ± 0.01a | 5628.38 ± 274.73a |
|  |  | CSFC | 5955.25 ± 344.15b | 4272.67 ± 228.48a | 9.79 ± 0.13a | 0.95 ± 0.01a | 5856.78 ± 573.09a |
|  |  | RCFC | 6515.29 ± 70.24a | 4585.33 ± 45.57a | 10.26 ± 0.08a | 0.95 ± 0.01a | 6009.89 ± 566.38a |
|  | **Budding** | CK | 4900.32 ± 159.15b | 3745.00 ± 366.79b | 8.89 ± 0.32b | 0.99 ± 0.02a | 4561.46 ± 273.23b |
|  |  | LSFC | 5236.21 ± 53.11a | 4312.13 ± 477.65a | 9.32 ± 0.35a | 0.98 ± 0.01a | 5468.03 ± 203.53a |
|  |  | RBFC | 5701.23 ± 226.78 | 4673.00 ± 200.19a | 10.28 ± 0.15a | 0.97 ± 0.01a | 6125.35 ± 602.03a |
|  |  | CSFC | 5552.16 ± 177.56a | 4574.49 ± 189.55a | 10.04 ± 0.23a | 0.97 ± 0.01a | 5156.04 ± 768.95a |
|  |  | RCFC | 5970.67 ± 400.19a | 4294.33 ± 189.49a | 10.18 ± 0.05a | 0.955 ± 0.01a | 5255.18 ± 677.13a |
|  | **Harvesting** | CK | 4549.61 ± 258.89c | 3504.33 ± 187.11c | 9.17 ± 0.09a | 0.99 ± 0.10a | 4560.14 ± 237.09c |
|  |  | LSFC | 5289.25 ± 311.31b | 4333.26 ± 267.47b | 9.75 ± 0.16a | 0.95 ± 0.01a | 5524.143 ± 349.73b |
|  |  | RBFC | 5202.46 ± 260.13b | 5025.33 ± 128.86a | 10.48 ± 0.01a | 0.96 ± 0.01a | 6794.86 ± 604.49a |
|  |  | CSFC | 5616.68 ± 688.54a | 4595.67 ± 161.86b | 10.23 ± 0.11a | 0.95 ± 0.01a | 5865.73 ± 278.52b |
|  |  | RCFC | 6172.52 ± 101.32a | 4482.00 ± 32.19b | 10.22 ± 0.03a | 0.92 ± 0.01a | 5509.91 ± 550.01b |

**Table S3.** Pearson correlation analysis between alpha diversity and disease incidence of tobacco bacterial wilt. (* and ** indicate correlation is significant at *p* < 0.05 and *p* < 0.01.)

|  | **OTUs** | **observed_species**  **(Sobs)** | **Shannon index** | **Simpson index** | **Chao1 index** |
| --- | --- | --- | --- | --- | --- |
| **Pearson** | −0.558** | −0.349* | −0.215 | −0.380* | −0.260 |
| ***p* value** | 0.000 | 0.019 | 0.156 | 0.010 | 0.085 |

**Table S4.** Pearson correlation analysis between top ten abundant bacterial phyla and disease incidence of tobacco bacterial wilt. (* and ** indicate correlation is significant at *p* < 0.05 and *p* < 0.01.)

|  | ***Proteobacteria*** | ***Acidobacteria*** | ***Actinobacteria*** | ***Chloroflexi*** | ***Gemmatimonadetes*** |
| --- | --- | --- | --- | --- | --- |
| **Pearson** | 0.137 | −0.190 | −0.118 | −0.627** | 0.535** |
| ***p* value** | 0.369 | 0.212 | 0.441 | 0.000 | 0.001 |
|  | ***Firmicutes*** | ***Bacteroidetes*** | ***Planctomycetes*** | ***Verrucomicrobia*** | ***Nitrospirae*** |
| **Pearson** | −0.455** | 0.092 | −0.467** | 0.414* | −0.858** |
| ***p* value** | 0.008 | 0.612 | 0.006 | 0.017 | 0.000 |

**Table S5.** Pearson correlation analysis between the disease incidence of tobacco bacterial wilt, bacterial community and the abundance of *R. solanacearum*. (** indicate correlation is significant at *p* < 0.01.**)**

|  | **2017** | | **2018** | | **2019** | |
| --- | --- | --- | --- | --- | --- | --- |
|  | **Pearson** | ***P*** | **Pearson** | ***P*** | **Pearson** | ***P*** |
| **Disease incidence of TBW** | 0.894** | 0.001 | 0.853** | 0.001 | 0.924** | 0.001 |
| **Bacterial community** | 0.029 | 0.863 | 0.124 | 0.492 | 0.015 | 0.931 |

**Table S6.** Monte Carlo test of the impact of soil physicochemical properties on the bacterial community structure. (*, ** and *** indicate correlation is significant at *p* < 0.05, *p* < 0.01 and *p* < 0.001.)

| **Variables** | **2017** | | **2018** | | **2019** | |
| --- | --- | --- | --- | --- | --- | --- |
|  | ***R^2^*** | ***P*** | ***R^2^*** | ***P*** | ***R^2^*** | ***P*** |
| **pH** | 0.548 | 0.001*** | 0.698 | 0.001*** | 0.541 | 0.001*** |
| **alkaline N** | 0.243 | 0.039* | 0.390 | 0.01** | 0.417 | 0.003** |
| **available P** | 0.375 | 0.005** | 0.467 | 0.001*** | 0.387 | 0.02** |
| **available K** | 0.191 | 0.082 | 0.243 | 0.039* | 0.242 | 0.032* |
| **organic matter** | 0.050 | 0.503 | 0.283 | 0.043* | 0.288 | 0.039* |
| **exchangeable Ca** | 0.081 | 0.336 | 0.041 | 0.626 | 0.049 | 0.526 |
| **exchangeable Mg** | 0.010 | 0.898 | 0.225 | 0.055 | 0.050 | 0.503 |
| **total N** | 0.054 | 0.571 | 0.106 | 0.281 | 0.080 | 0.349 |

**Table S7.** Pearson correlation analysis between beneficial bacterial and disease incidence of tobacco bacterial wilt. (* and ** indicate correlation is significant at *p* < 0.05 and *p* < 0.01.)

|  | ***Acinetobacter*** | ***Azospirillum*** | ***Bradyrhizobium*** | ***Chthonomonas*** | ***Granulicella*** |
| --- | --- | --- | --- | --- | --- |
| **Pearson** | −0.899** | −0.873** | −0.886** | −0.847** | −0.863** |
| ***p* value** | 0.000 | 0.000 | 0.000 | 0.000 | 0.000 |
|  | ***Hyphomicrobium*** | ***Lysobacter*** | ***Mesorhizobium*** | ***Methylobacterium*** | ***Microbispora*** |
| **Pearson** | −0.837** | −0.012 | −0.905** | −0.284 | 0.845** |
| ***p* value** | 0.000 | 0.967 | 0.000 | 0.305 | 0.000 |
|  | ***Microvirga*** | ***Novosphingobium*** | ***Pseudomonas*** | ***Psychrobacter*** | ***Rhizobium*** |
| **Pearson** | 0.718** | 0.500 | −0.578* | −0.796** | −0.421 |
| ***p* value** | 0.003 | 0.057 | 0.024 | 0.000 | 0.118 |
|  | ***Rhodococcus*** | ***Solirubrobacter*** | ***Stenotrophomonas*** | ***Steroidobacter*** | ***Gaiellales*** |
| **Pearson** | 0.116 | 0.173 | −0.660** | −0.199 | 0.853** |
| ***p* value** | 0.681 | 0.537 | 0.007 | 0.478 | 0.000 |

**Table S8.** Pearson correlation analysis between antagonistic bacterial and disease incidence of tobacco bacterial wilt. (* and ** indicate correlation is significant at *p* < 0.05 and *p* < 0.01.)

|  | ***Actinospica*** | ***Bacillus*** | ***Burkholderia*** | ***Catenulispora*** | ***Chryseobacterium*** |
| --- | --- | --- | --- | --- | --- |
| **Pearson** | −0.738** | −0.740** | −0.791** | −0.613* | 0.318 |
| ***p* value** | 0.002 | 0.002 | 0.000 | 0.015 | 0.248 |
|  | ***Clostridium*** | ***Flavobacterium*** | ***Haliangium*** | ***Microbispora*** | ***Paenibacillus*** |
| **Pearson** | 0.148 | 0.135 | 0.708** | −0.548* | −0.029 |
| ***p* value** | 0.600 | 0.632 | 0.003 | 0.035 | 0.919 |
|  | ***Rhodopseudomonas*** | ***Sphingomonas*** | ***Staphylococcus*** | ***Stenotrophomonas*** | ***Streptomyces*** |
| **Pearson** | −0.454 | 0.096 | 0.061 | 0.022 | 0.164 |
| ***p* value** | 0.089 | 0.733 | 0.828 | 0.937 | 0.560 |
